# Metabolic substrate utilization in stress-induced immune cells

**DOI:** 10.1186/s40635-020-00316-0

**Published:** 2020-12-18

**Authors:** Xiaomin Zhang, Fabian Zink, Felix Hezel, Josef Vogt, Ulrich Wachter, Martin Wepler, Maurizio Loconte, Christine Kranz, Andreas Hellmann, Boris Mizaikoff, Peter Radermacher, Clair Hartmann

**Affiliations:** 1 Institut für Anästhesiologische Pathophysiologie und Verfahrensentwicklung, Universitätsklinikum Ulm, Helmholzstraße 8/1, 89081 Ulm, Germany; 2 Klinik für Anästhesiologie, Universitätsklinikum Ulm, Ulm, Germany; 3 Anesthesia and Intensive Care, San Martino Policlinico Hospital, IRCCS for Oncolocy and Neuroscience, Genoa, Italy; 4 Institut für Analytische und Bioanalytische Chemie, Universität Ulm, Ulm, Germany

**Keywords:** Immunometabolism, Glycolysis, Pentose phosphate pathway, Tricarboxylic acid cycle, Oxidative phosphorylation, Reactive oxygen species, Catecholamines

## Abstract

Immune cell activation leads to the acquisition of new functions, such as proliferation, chemotaxis, and cytokine production. These functional changes require continuous metabolic adaption in order to sustain ATP homeostasis for sufficient host defense. The bioenergetic demands are usually met by the interconnected metabolic pathways glycolysis, TCA cycle, and oxidative phosphorylation. Apart from glucose, other sources, such as fatty acids and glutamine, are able to fuel the TCA cycle.

Rising evidence has shown that cellular metabolism has a direct effect on the regulation of immune cell functions. Thus, quiescent immune cells maintain a basal metabolic state, which shifts to an accelerated metabolic level upon immune cell activation in order to promote key effector functions.

This review article summarizes distinct metabolic signatures of key immune cell subsets from quiescence to activation and demonstrates a methodical concept of how to assess cellular metabolic pathways. It further discusses why metabolic functions are of rising interest for translational research and how they can be affected by the underlying pathophysiological condition and/or therapeutic interventions.

## Introduction

Immune cell responses in inflammation are highly dynamic and require continuous metabolic adaption in order to maintain a sufficient host defense. The bioenergetic demands are usually met by the interconnected metabolic pathways glycolysis, tricarboxylic acid (TCA) cycle, and oxidative phosphorylation (OXPHOS). Briefly, during glycolysis, glucose is converted into pyruvate in the cytoplasm and enters the mitochondria after the conversion to acetyl-coenzyme A (-CoA). In the TCA cycle, acetyl-CoA is degraded, resulting in nicotinamide adenine dinucleotide (NADH) and flavin adenine dinucleotide (FADH_2_) formation, which serve as electron donors for OXPHOS, ultimately resulting in adenosine triphosphate (ATP) generation. As a natural byproduct of the electron transport within the respiratory chain, reactive oxygen species (ROS) are produced alongside OXPHOS. Apart from glucose, other sources, such as fatty acids and glutamine, are able to fuel the TCA cycle. Fatty acids enter the TCA cycle after being subjected to β-oxidation to generate acetyl-CoA. Glutamine is converted to glutamate via glutaminolysis and enters the TCA cycle after being transformed to α-ketoglutarate.

**Fig. 1:**
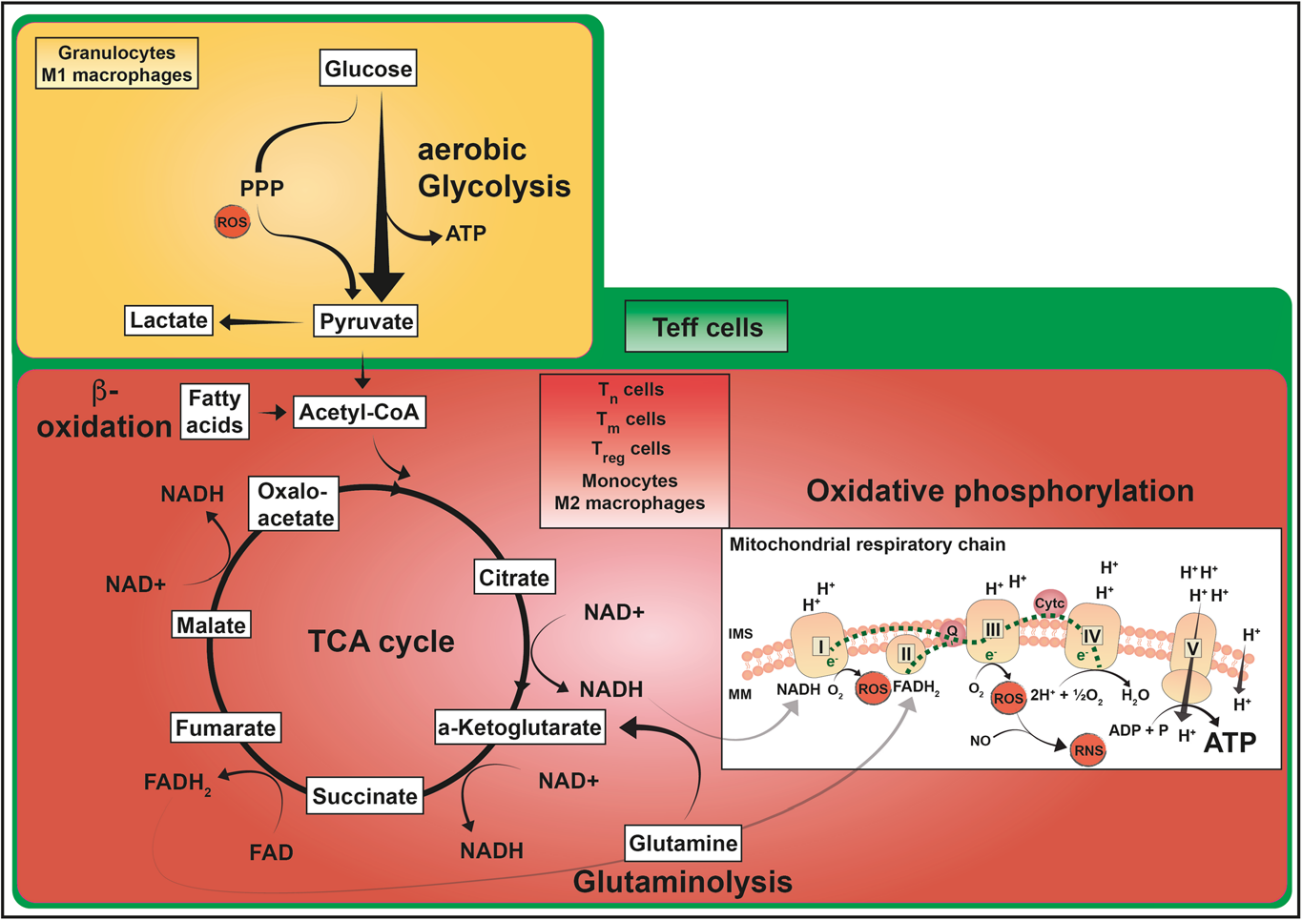
ATP-producing metabolic pathways in distinct immune cell subsets. Glucose oxidation to pyruvate via glycolysis is a fast reaction generating 2 mol of ATP per mol glucose. This aerobic glycolysis is complemented by the PPP that can produce further metabolic precursor molecules and is involved in ROS production. Pyruvate can be converted to lactate or can be further oxidized to acetyl-CoA entering the mitochondrial TCA cycle (yellow box). The TCA cycle (red box) generates reducing equivalents NADH and FADH_2_ which are utilized in the mitochondrial respiratory chain to build up the proton gradient across the mitochondrial inner membrane by complexes I–IV of the respiratory chain. As a by-product, ROS and RNS are produced. Oxidative phosphorylation produces larger amounts of ATP (36–38 mol/mol glucose) by complex V. Immune cells are also able to utilize substrates such as glutamine, which enters these pathways via the TCA metabolite α-ketoglutarate, and fatty acids, which are oxidized to acetyl-CoA via β-oxidation. Granulocytes and M1 macrophages have a highly glycolytic metabolism (yellow box) even when oxygen is available. Their TCA cycle and respiratory chain activity is kept at low level. T_n_, T_m_, and T_reg_ cells as well as monocytes and M2 macrophages primarily perform OXPHOS and are also able to metabolize fatty acids and glutamine in order to fuel the TCA cycle (red box). T_eff_ cells have a highly active metabolism including all of the pathways described (green box). These pathways do not only culminate in ATP production but also provide other biosynthetic pathways with metabolic precursors thus supporting different functional necessities of the immune cell populations. Abbreviations: ADP, adenosine diphosphate; ATP, adenosine triphosphate; CoA, coenzyme A; FADH_2_/FAD, flavin adenine dinucleotide in its reduced/oxidized form; IMS, intermembrane space; MM, mitochondrial membrane; NADH/NAD^+^, nicotinamide adenine dinucleotide in its reduced/oxidized form; OXPHOS, oxidative phosphorylation; PPP, pentose phosphate pathway; ROS, reactive oxygen species; RNS, reactive nitrogen species; TCA, tricarboxylic acid cycle; T_eff_, effector T cell; T_n_, naïve T cell; T_m_, memory T cell; T_reg_, regulatory T cell

It is well-known that under hypoxic conditions ATP is generated via anaerobic glycolysis by converting pyruvate to lactate. Intriguingly, some types of immune cells show a similar metabolism even when oxygen availability is not limited. These cells preferentially use aerobic glycolysis for ATP production, a metabolic pathway which was initially reported in cancer cells by Warburg et al. [1]. It is of course undisputable that, regarding the ATP yield, aerobic glycolysis (2 mol ATP/mol glucose) is far less efficient compared to OXPHOS (≈ 36 mol ATP/mol glucose); however, this difference can be compensated for by a high speed of aerobic glycolysis [2]. A high flux through aerobic glycolysis subsequently increases the flux through the pentose phosphate pathway (PPP), a metabolic shunt that parallels glycolysis. The PPP generates five-carbon sugars for nucleotide synthesis and nicotinamide adenine dinucleotide phosphate (NADPH), crucial for NADPH-dependent oxidative burst and fatty acid biosynthesis. Figure 1 summarizes core metabolic pathways of immune cell subsets, which will be discussed in the following manuscript.

As mentioned above, biological functions of immune cells are tightly linked to metabolic programs, which are highly plastic and adapt to promote their changing functions. This review article summarizes distinct metabolic signatures of key immune cells from quiescence to activation and discusses their potential role as bioenergetic biomarkers in translational research.

## Distinct energetic profiles of immune cells

In general, immune responses are composed of the innate and the adaptive immune system. Upon encountering with a pathogen, the innate immune response, which is composed of neutrophils, monocytes/macrophages, and natural killer (NK) cells, represents the first stage and is nonspecific and rapid. The second stage is defined by the adaptive branch of the immune response, which is composed of a small fraction of cells highly specific for any pathogen/antigen and comprises T cells and B cells. An immunological memory is not only known for the adaptive immune system but has previously also been attributed to the cells of the innate immune system, also referred to as trained immunity. Trained immunity is particularly triggered by pattern recognition receptors, which are expressed on innate immune cells in order to recognize pathogen molecules and is regulated by metabolic and epigenetic reprogramming [3, 4]. Thus, similarly to the known immune responses, trained immunity induces changes in intracellular immune signaling by rewiring the energy metabolism. This leads to an enhanced proinflammatory response upon a subsequent inflammatory challenge eventually improving survival of the host. In contrast to the adaptive immune system, innate immune responses are unspecific due to a lacking antibody-antigen interaction.

**Fig. 2:**
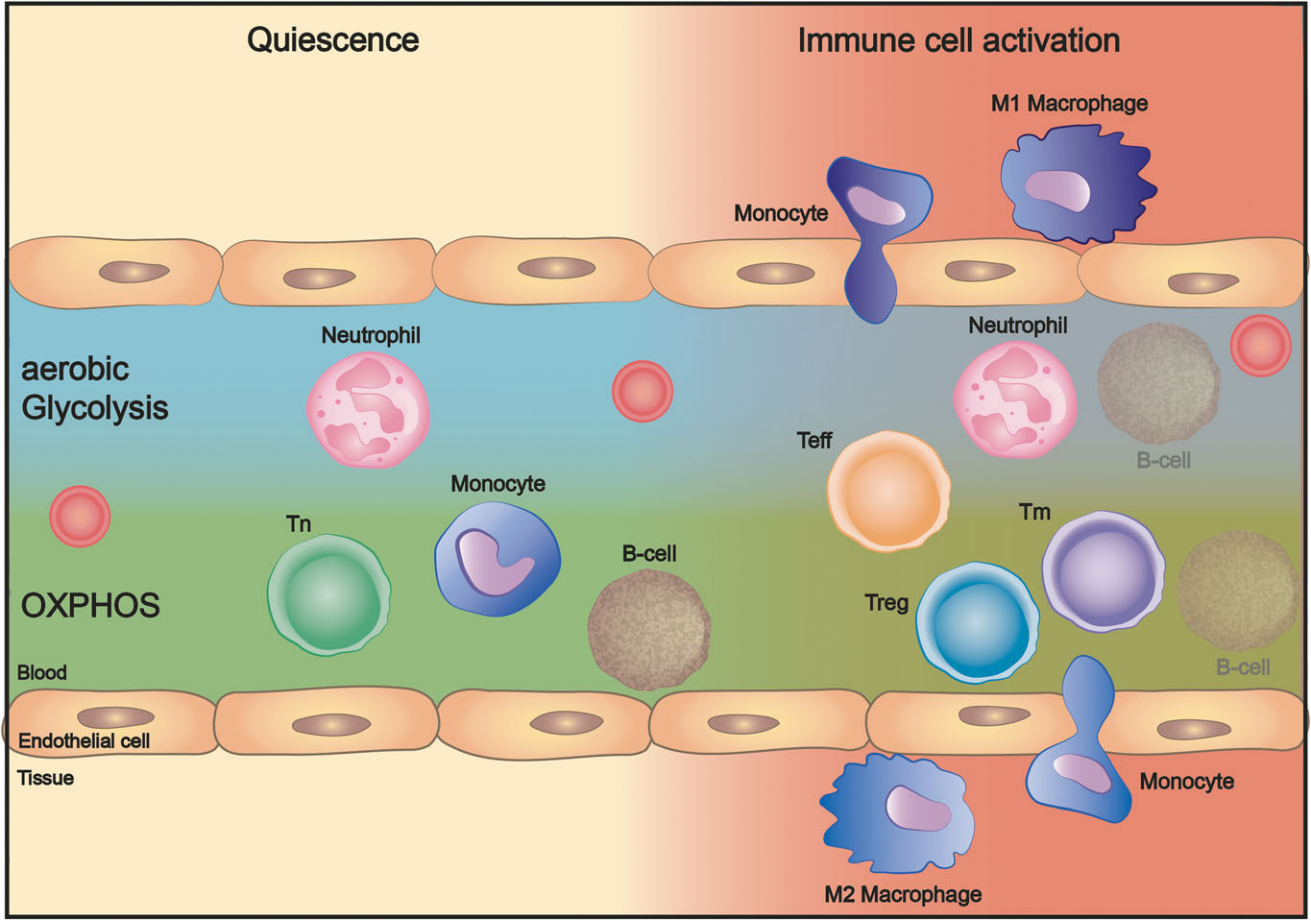
Metabolic pathways of distinct immune cell subsets in quiescence and upon activation. Under quiescent conditions, neutrophils rely on aerobic glycolysis, whereas T_n_ cells, monocytes, and B cells preferentially rely on OXPHOS for ATP production. Immune cell activation reshapes the metabolic demands of immune cells performing their various effector functions, differentiation to T_eff_, T_reg_, or T_m_ cells, and migration across the endothelium into the tissue, where substrate and oxygen availability can be limited. Abbreviations: ATP, adenosine triphosphate; OXPHOS, oxidative phosphorylation; T_eff_, effector T cell; T_n_, naïve T cell; T_m_, memory T cell; T_reg_, regulatory T cell

Regarding the energy metabolism, quiescent immune cells maintain a basal metabolic state, which shifts to a higher metabolic level upon immune cell activation in order to promote key effector functions. The cell type-specific differences of ATP-producing pathways in quiescence and upon activation will be outlined below (Fig. 2).

### Granulocytes

Neutrophils only have a few mitochondria, primarily important for mitochondrial membrane potential maintenance and apoptosis initiation [5, 6]. Neutrophils consume low amounts of oxygen [7] and produce ATP through aerobic glycolysis [8–10]. Metabolic profiles of granulocytes have mostly been described in neutrophils; however, there is some evidence showing that basophilic and eosinophilic granulocytes depend on glycolytic metabolism [11]. Besides, Porter et al. demonstrated that human eosinophils exhibited a higher basal oxygen consumption rate and reserve respiratory capacity, thereby allowing them to be metabolically more versatile [12].

After the recruitment to an inflammatory tissue site in response to pro-inflammatory stimuli, neutrophils become fully activated, a condition of diverse functions including oxidative burst, the production of neutrophil extracellular traps (NETs), and phagocytosis. In this state, neutrophils require large amounts of ATP, which is primarily produced via aerobic glycolysis [13, 14]. This oxygen-independent metabolism is particularly beneficial when neutrophils are recruited to inflammatory sites with low oxygen partial pressure.

Hypoxia and inflammation are tightly related: hypoxia may be the consequence of inflammatory processes or may vice versa actively promote inflammatory responses. In both cases, it is necessary that immune cells sense and adapt to low oxygen levels in order to continue to perform their cell type-specific functions under these conditions. The metabolic adaption to hypoxia is primarily mediated by hypoxia-inducible transcription factors (HIFs), a family of transcription factors with oxygen-sensitive α-subunits. However, HIFs can also be activated by an inflammatory reaction thereby upregulating glycolytic gene expression to safeguard elevated ATP requirements via aerobic glycolysis and by generating valuable precursors for cellular proliferation and effector function, e.g., nucleotides, lipids, and amino acids [15–17]. The effects of HIFs strongly depend on the cell type. In myeloid cells, which are primarily responsible for innate immune defenses, HIF1α promotes pro-inflammatory effects by increasing pathogen clearance features, such as motility, invasiveness, and bactericidal activity [18]. In the adaptive immune system, HIF1α promotes the differentiation of regulatory T cells (T_reg_), thereby increasing anti-inflammatory and tissue-protective effects [19]. Thus, HIF1α specifically modulates the metabolism of distinct immune cell subsets in order to generate a balanced and controlled immune response [20].

In neutrophils, adequate intake of glucose is particularly mediated by the glucose transporter-1 (GLUT-1) and GLUT-3 [21], while HIF1α maintains glycolysis by upregulating genes of key glycolytic enzymes and at the same time repressing genes for OXPHOS [18]. Glycolysis is crucial for neutrophilic functions, such as phagocytosis and NET formation, a mechanism to kill microorganisms by releasing chromatin traps, as shown in phorbol myristate acetate (PMA)-stimulated human neutrophils [22]. Furthermore, other authors highlighted the importance of the PPP in NET formation, arising mainly from NADPH oxidase (NOX)-derived ROS and further highlighted the importance of mammalian target of rapamycin (mTOR) regulating HIF1α in this context [23, 24].

Upon neutrophil activation, a high flux through the glycolytic pathway subsequently increases the flux through the PPP, in order to generate NADPH, which is in turn used by NOX to generate superoxide anions (O_2_^−^) [25–27]. This mechanism is also referred to as the “oxidative burst.” Guthrie et al. demonstrated that human lipopolysaccharide (LPS)-stimulated neutrophils increased their glucose uptake and oxygen consumption, which, however, was used for hydrogen peroxide (H_2_O_2_) production rather than OXPHOS [28].

Last, due to their metabolic plasticity, neutrophils are able to meet their energy requirements during differentiation processes and/or under glucose-limiting conditions by utilizing other metabolic substrates, such as fatty acids via β-oxidation and glutamine via glutaminolysis [29–31].

### Monocytes

Monocytes are crucial effectors and regulators of innate immune response. Resting monocytes do not proliferate and remain in a low metabolic steady state driven by oxidative metabolism [32].

Upon an inflammatory stimulus, activated monocytes eliminate pathogens via phagocytosis, ROS, and cytokine production, among others, by shifting their energy metabolism to a glycolytic phenotype, mediated by mTOR-HIF1α, in order to satisfy higher ATP requirements [33, 34]. Besides, monocytes are recruited to the inflammatory tissue and continue to differentiate into two different subtypes: M1 macrophages, promoting a pro-inflammatory response [35], or M2 macrophages, which are anti-inflammatory and play a vital role in modulating inflammation and repairing tissue [36].

These highly distinct functional phenotypes are reflected by opposing metabolic requirements. The pro-inflammatory M1 condition depends on aerobic glycolysis and thus shows low oxygen consumption rates [34, 37, 38]. Glucose uptake is enhanced by the increased expression of GLUT1-3 as demonstrated in PMA- or LPS-stimulated human monocytes, but also in T and B lymphocytes [21]. Similarly, as shown in neutrophils, HIF1α is crucially involved in promoting/maintaining a glycolytic metabolism [39]. However, macrophages are not only able to utilize glucose but also glutamine as demonstrated in activated murine peritoneal macrophages by Newsholme et al. [40].

During the resolution of inflammation, macrophages differentiate into the M2 phenotype, which primarily performs OXPHOS metabolism fueled by fatty acid oxidation (FAO) [41]. In fact, after inhibition of OXPHOS, M2 macrophage expression was attenuated, and, moreover, they were forced into the M1 macrophage phenotype [32, 42]. In other words, the functional (M1 vs. M2) state of monocytes coincides with a time-dependent sequence of metabolic activity, with enhanced glycolysis and PPP turnover during the activation phase and back to TCA and OXPHOS, respectively, during the deactivation phase i.e. the resolution phase of inflammation [43, 44].

Monocytes have extensively been studied in respect to trained immunity. Arts et al. elegantly demonstrated that β-glucan-induced trained immunity in human monocytes was mediated by profound rewiring of cellular metabolism [45]. Thus, particularly glycolysis, glutaminolysis and cholesterol synthesis were described as key metabolic pathways. Besides, the authors identified the TCA metabolite fumarate as a crucial player in promoting epigenetic reprogramming [45]. Similarly, other studies in human β-glucan trained monocytes reported an increase of particularly glycolytic genes primarily mediated by the Akt/mTOR/HIF1α pathway [46].

### Lymphocytes

#### T lymphocytes

Unstimulated naïve T lymphocytes primarily use OXPHOS to generate ATP [10, 47]. Subsequently to antigen recognition and co-stimulation, activated T lymphocytes rapidly grow and differentiate into subpopulations, such as effector T cells (T_eff_), T_reg_, and memory T cells (T_m_). This developmental program requires large amounts of energy in order to generate a sufficient amount of ATP [48–50]. T_eff_ cells are crucial players during an inflammatory response. They have both immune promoting but also negative regulatory effects thereby steering immune responses. T_eff_ cells have a reduced mitochondrial mass and a low reserve respiratory capacity and generate ATP predominantly through aerobic glycolysis over OXPHOS [51]. This coincides with increased GLUT-1 expression and glucose uptake [52–55]. Apart from a high glycolytic rate, T_eff_ show increased biosynthetic activity by promoting nucleotide synthesis via PPP.

In contrast, T_reg_ cells and T_m_ cells are non-proliferative cells and adopt OXPHOS and FAO for ATP generation [56–58]. T_reg_ cells are a specialized subpopulation of lymphocytes that suppress immune responses to balance pro-inflammation. According to Angelin et al., T_reg_ cells adopt oxidative metabolism induced by forkhead box protein 3 (FoxP3), which can downregulate glycolysis in mouse models including colitis, cardiac allografting, and homeostatic proliferation [59]. Consistent with this notion, Gerriets et al. found T_reg_ cells to also display aerobic glycolysis when FoxP3 is reduced in a murine model [60]. T_m_ embody features of both naive and effector cells_._ Bioenergetically, T_m_ cells have more mitochondrial mass and a higher reserve respiratory capacity compared to naïve cells [61]. They are metabolically primed and thus are able to rapidly respond when the same pathogen attacks the host [57].

#### B lymphocytes

B cells are a critical part of the humoral immunity e.g. by secreting antibodies and promote T cell activation [62]. However, data on B cell lymphocyte metabolism is relatively scarce, as the previously mentioned studies primarily concentrated on T lymphocytes. Recent evidence showed that resting B cells seem to have lower energy requirements than resting T cells as they consumed less glucose and fatty acids and, consequently, produced less ATP [63]. Nevertheless, resting B cells primarily rely on OXPHOS to meet their metabolic demands and have a higher mitochondrial mass [63]. Despite these differences in the resting state, B cells share some metabolic characteristics with T cells upon activation. Thus, these cells rapidly increase glucose uptake following cell antigen receptor activation; phosphatidylinositol 3-kinase (PI-3 K) is indispensable to increase glucose utilization [64, 65]. In line with this, Limon et al. showed that B cells largely depend on glycolysis for proliferation [66]. However, recent research from Waters et al. displayed different notions that activated B cells upregulated OXPHOS rather than glycolysis, despite increased glucose uptake during B cell activation [67].

## A concept to assess immune cell metabolism

**Fig. 3:**
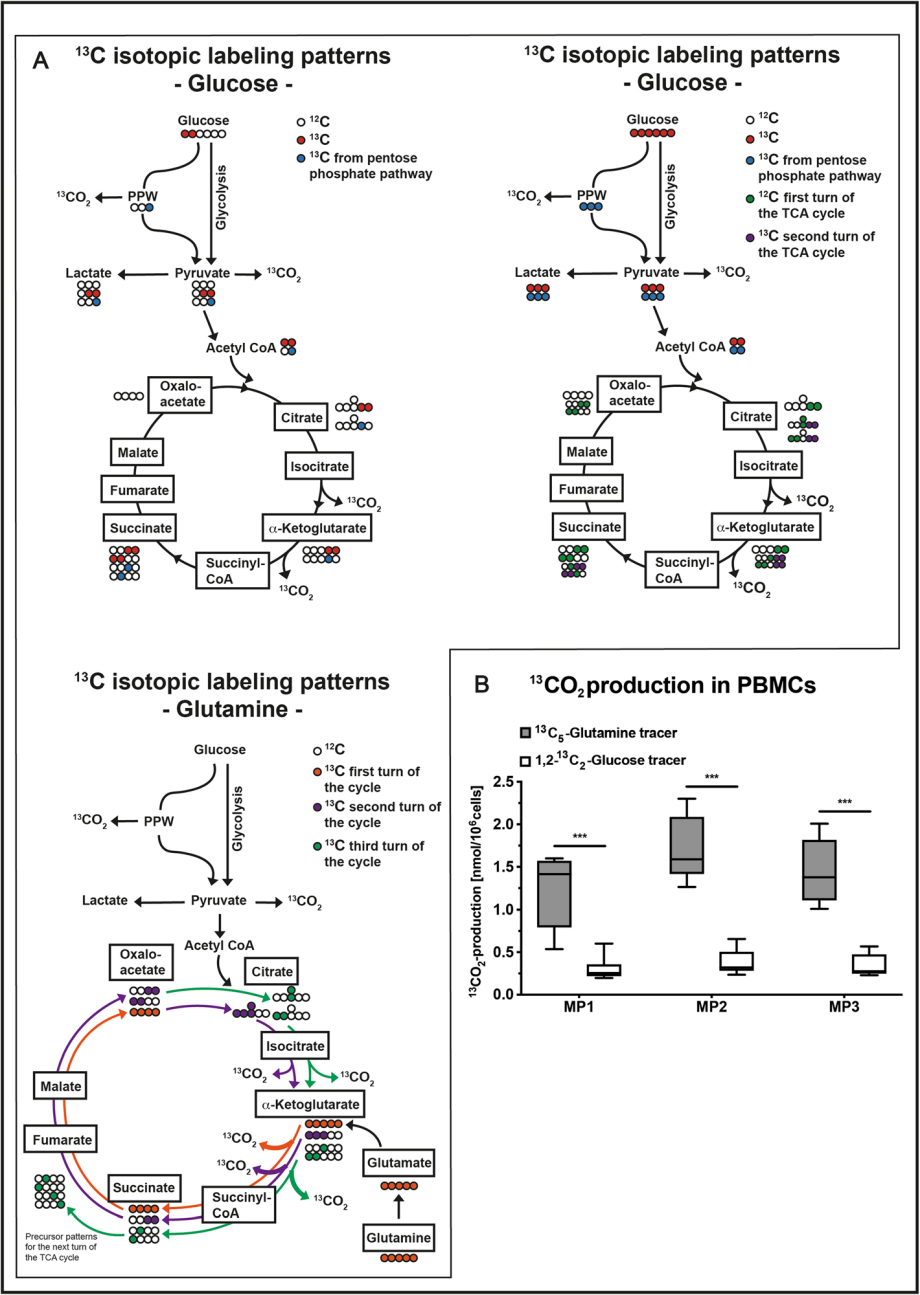
Assessing cellular metabolism with stable isotope-labeled substrates. **a** ^13^C labeling patterns of TCA cycle metabolites resulting from utilization of 1,2-^13^C_2_-glucose (upper left), ^13^C_6_-glucose (upper right), and ^13^C_5_-glutamine (lower left). Depending on the substrate used, further conclusions can be drawn on the involvement of the PPP or the cycling within the TCA cycle. **b** ^13^CO_2_ production in PBMCs from a porcine model of acute subdural hematoma at MP before (MP1), 12 h (MP2), and 24 h (MP3) after hematoma induction. 5 × 10^6^ PBMCs/mL were incubated in chemically identical RPMI media containing either 1,2-^13^C_2_-glucose, ^13^C_6_-glucose, or ^13^C_5_-glutamine. Supernatant was transferred to airtight vials and acidified to release CO_2_ into the gas phase where ^13^CO_2_ enrichment was determined by gas chromatography-mass spectrometry. Data are presented as median and interquartile range; ***p < 0.0001 according to a 2-way ANOVA and a Sidak’s multiple comparisons test, *n* = 5–6. Abbreviations: CO_2_, carbon dioxide; MP, measurement timepoint; PBMCs, peripheral blood mononuclear cells; PPP, pentose phosphate pathway; TCA, tricarboxylic acid cycle

**Fig. 4:**
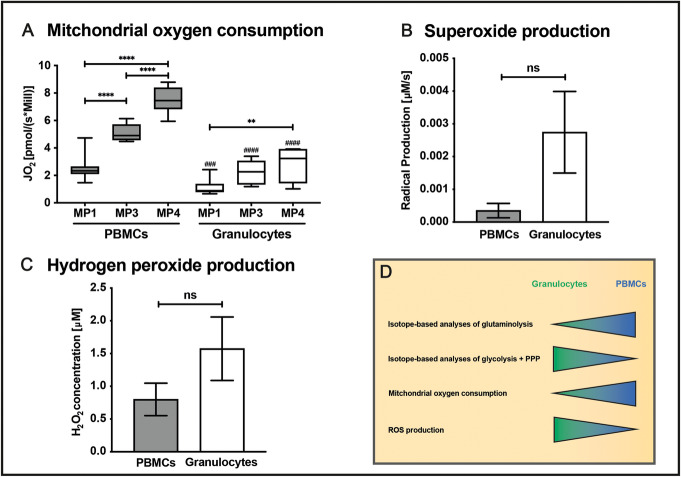
Assessment of immune cell metabolism. Panel **a** shows the mitochondrial oxygen consumption in a porcine model of acute subdural hematoma combined with hemorrhagic shock assessed in the uncoupled state (electron transport system capacity) before (MP1), as well as 12 (MP2) and 24 h (MP3) after trauma induction in PBMCs (gray bars) and granulocytes (white bars). 10 × 10^6^ cells/1 mL RPMI medium were added to the measurement chamber of the Oxygraph O2K (Oroboros Instruments, Innsbruck, Austria) which measures oxygen concentration with a Clark electrode. After addition of the ATP synthase inhibitor oligomycine (0.5 μL of a 0.5 μM stock solution), the uncoupling agent FCCP (0.5 μL of a 0.5 μM stock solution) was added stepwise to reach the level of maximum oxygen consumption (electron transfer system capacity). **b** O_2_^−^ production quantified by electron spin resonance spectroscopy in PBMCs (gray bars) and granulocytes (white bars) isolated from pigs before trauma induction. Therefore, 2.5 × 10^6^ cells (in 1 mL RPMI) were mixed with the superoxide-targeted spin probe CMH. A serial measurement over 30 min enables calculation of superoxide radical production rate. An equally treated sample of RPMI with CMH served as blank value that was subtracted from the cell suspensions’ production rate. **c** Before trauma induction, H_2_O_2_ production of 1 × 10^6^ PBMCs (gray bars) and granulocytes (white bars) in 2 mL RPMI medium was electrochemically quantified with a Pt-black modified microelectrode. The data presented in **b** and **c** are adapted from [72]. **d** outlines the concept for our methodical approach to analyze the energy metabolism in granulocytes and PBMCs. Usually, granulocytes preferentially utilize glucose to produce ATP and have a low mitochondrial oxygen consumption but a high ROS production. PBMCs on the other hand prefer glutamine over glucose utilization, have a higher mitochondrial oxygen consumption and low ROS production. It is important to note that the metabolic pathways shift depending on the state of activation and that immune cells use different substrates (fatty acids, amino acids) in order to safeguard ATP homeostasis. Data are presented as **a** median with interquartile range and **b**, **c** mean with SD, *p < 0.05; **p < 0.01; ***p < 0.001; ****p < 0.0001; ^###^*p* < 0.001 vs PBMC; ^####^*p* < 0.0001 vs PBMC; according to a **a** 1-way ANOVA and Tukey’s multiple comparisons test, *n* = 6–14, and **b** Wilcoxon signed-rank test, *n* = 7–9, **c** *n* = 3 per group. Abbreviations: ATP, adenosine triphosphate; CMH, 1-hydroxy-3-methoxycarbonyl-2,2,5,5-tetramethylpyrrolidine (spin probe); FCCP, carbonyl cyanide-4-(trifluoromethoxy)phenylhydrazone (uncoupling agent); MP, measurement timepoint; PBMCs, peripheral blood mononuclear cells; Pt-black, platinum black; ROS, reactive oxygen species; SD, standard deviation

The variable substrate utilization of immune cells as well as their impact on energy metabolism can be assessed ex vivo by the following methodical approach: (1) Metabolic flux analysis is performed by incubating immune cells with stable, non-radioactive isotope-labeled substrates (e.g., parallel incubation with ^13^C-glucose, ^13^C-glutamine [68]). Subsequently, measurements of the isotope enrichment in various metabolites and cleavage products of the glycolytic pathway, the PPP, and the TCA-cycle are performed, followed by conversion of labeling patterns to estimate relative pathway activities and by quantification of ^13^CO_2_ release from the respective isotopes (Fig. 3) [69–71]. (2) Measurement of mitochondrial oxygen consumption and (3) production of ROS (Fig. 4).

We recently showed in a long-term, resuscitated porcine model of acute subdural hematoma (ASDH) combined with hemorrhagic shock that the mitochondrial oxygen consumption was more pronounced in peripheral blood mononuclear cells (PBMCs) than in granulocytes in both the quiescent (MP1, measuring point) and the activation state (MP3 + 4) (Fig. 4a, unpublished data). In contrast, granulocytes had a several-fold higher production of both the O_2_^−^ (Fig. 4b, adapted from [72]**)** and the reactive species H_2_O_2_ (Fig. 4c, adapted from [72]**)**. In respect to the isotope data, we could recently demonstrate in a long-term, resuscitated porcine ASDH-induced acute brain injury model [73] that PBMC-related ^13^CO_2_ production from glutamine was approximately five times higher than that of glucose-derived ^13^CO_2_ (Fig. 3b, unpublished data). In line with our findings, Fig. 4d reflects the concept for this methodical approach of analyzing the energy metabolism in distinct immune cell populations. According to their ability of metabolizing glutamine (glutaminolysis), ^13^CO_2_ production from glutamine is higher in PBMCs compared to ^13^CO_2_ derived from glucose, due to their low rate of aerobic glycolysis (Fig. 4d). PBMCs primarily perform OXPHOS in order to produce ATP, which is reflected by a high rate of mitochondrial oxygen consumption. In contrast, granulocytes generate ATP via aerobic glycolysis, resulting in low oxygen consumption rates (Fig. 4d). A high flux through the glycolysis pathway is accompanied by a high flux through the PPP leading to higher production rates of ROS in granulocytes compared to PBMCs (Fig. 4d).

## The role of reactive oxygen species and catecholamines

As mentioned above, ROS are a natural byproduct of the electron transport within the respiratory chain. Although early literature from Boveris et al. estimated that about 1–2% of the total consumed oxygen was diverted to H_2_O_2_ production in a steady state [74], recent findings take a more comprehensive approach into account. Thus, the rate of mitochondrial O_2_^−^ and H_2_O_2_ production depends on the cell type, on substrate availability, the source of reducing equivalents, and on (patho-)physiological states [75, 76].

ROS production is crucial for both signaling and host defense [77–79]. Grondman et al. demonstrated this crucial role of ROS in human monocytes: ROS production was directly correlated to the percentage of microbial killing of *Candida albicans* [80]. The authors elegantly showed that enhanced ROS production was increased due to metabolic changes e.g. increased aerobic glycolysis, PPP, and oxidative burst. In monocytes of healthy volunteers intravenously challenged with LPS to simulate sepsis-induced immunoparalysis, killing capacity was reduced, which coincided with impaired ROS production and less marked metabolic changes [80, 81]. In other words, impaired monocyte “metabolic plasticity” following endotoxin-induced immunotolerance lead to decreased ROS release and, consecutively, impaired host defense capacity. As a result of the abovementioned difference in mitochondria content, granulocytes and monocytes not only show markedly different respiratory activity, but also ROS formation. Clearly, despite its fundamental role for host defense, increased granulocyte-derived ROS production may also have a “dark side”: Kramer et al. reported in activated neutrophils that NADPH oxidase-derived H_2_O_2_ inhibited the metabolic shift of lymphocytes from OXPHOS to aerobic glycolysis, which was associated with decreased cytokine production as a mirror of depressed T_eff_ function [82].

Catecholamines have a profound impact on immune cell function [83, 84]. Their effect is dependent on the respective receptor stimulation: e.g., noradrenaline, the drug of choice for first-line hemodynamic support during septic shock, displays a pro-inflammatory profile when acting on α-adrenoceptors by upregulating NF-κB, while β-adrenoceptor activation results in a more immunosuppressive pattern [84]. Vice versa, cells of the innate immune system, such as granulocytes and macrophages, are able to produce catecholamines, which may per se aggravate inflammatory responses [85].

Apart from their direct role on modulating inflammatory processes, catecholamines have also been associated with enhanced oxidative stress levels due to autooxidation [86, 87]. It is known from the literature that increased radical production can impair mitochondrial oxygen uptake. In line with this, Lünemann et al. showed that noradrenaline dose-dependently exerted anti-inflammatory effects by inhibiting mitochondrial function of PBMCs obtained from healthy blood donors [88].

## Immune cell bioenergetics and outcome

Several authors showed in various pathological conditions that any impairment of immune cell oxygen consumption coincides with aggravated morbidity and mortality: Belikova et al. compared OXPHOS of PBMCs taken from healthy volunteers and patients with severe sepsis [89]. OXPHOS was higher in naïve PBMCs from patients with sepsis, but due to impaired responsiveness, this result was reversed upon stimulation with adenosine diphosphate (ADP). Incubation of healthy volunteer PBMCs with plasma from septic patients mimicked this finding [89]. In addition, Li et al. showed that mitochondrial oxygen consumption was reduced in PBMCs from patients with early-stage heart failure when compared to healthy volunteers [90], and Weiss et al. demonstrated mitochondrial dysfunction in circulating PBMCs during pediatric septic shock [91]. Cheng et al. used several approaches in order to assess the energy metabolism of immune cells in critical illness [92]. During the acute phase of the infection, the mTOR pathway orchestrated a shift from OXPHOS to glycolysis in PBMCs stimulated with either *C. albicans* or LPS (*E. coli*). In another approach, leukocytes obtained from either septic patients or healthy volunteers undergoing experimental endotoxemia showed a strong impairment of the cellular energy metabolism (glycolysis, mTOR signaling, OXPHOS, and FAO), that coincided with a decreased capacity to respond to secondary infection, also referred to as immunometabolic paralysis. This effect was partly reversed by the administration of interferon (INF)-γ, thereby underpinning its use in the treatment of sepsis [92].

In line with the abovementioned study, the assessment of energy metabolism of circulating immune cells may in fact provide a valuable approach for translational research, thereby modulating inflammatory responses or serving as a bioenergetic biomarker. However, before the energy metabolism can reliably be linked to pathophysiological states, more research has to be performed, particularly by precisely characterizing the energy metabolism of distinct subsets through more sophisticated approaches.

## Conclusion

During an inflammatory response, immune cells are activated, undergo proliferation and differentiation, and exert key effector functions. This requires a continuous metabolic adaption.

Immune cell functions are tightly linked to metabolic programs and demonstrate metabolic alterations upon immune cell activation. Thus, inactivated immune cells are metabolically quiescent and shift to a higher metabolic level upon an immunological challenge. Immune cell energy metabolism can be further influenced by various factors: inflammatory responses can increase intracellular ROS levels. ROS are essential for various biological functions, such as reactive oxygen burst, however, also affect the energy metabolism. Besides, immune cell metabolism can be affected by a common therapeutic intervention, such as catecholamines, thereby reinforcing pro- or anti-inflammatory responses.

## Data Availability

Data sharing is not applicable to this article as no datasets were generated or analyzed during the current study.

## Abbreviations

ADP/ATP: Adenosine di-/triphosphate
CO_2_: Carbon dioxide
CoA: Coenzyme A
DC: Dendritic cell
FADH_2_: Flavin adenine dinucleotide
FAO: Fatty acid oxidation
GLUT: Glucose transporter
HIF1α: Hypoxia-induced factor 1α
H_2_O_2_: Hydrogen peroxide
IFN: Interferon
LPS: Lipopolysaccharide
mTOR: Mammalian target of rapamycin
NADH: Nicotinamide adenine dinucleotide
NADPH: Nicotinamide adenine dinucleotide phosphate
NETs: Neutrophil extracellular traps
NOX: NADPH oxidase
O_2_^−^: Superoxide anion
OXPHOS: Oxidative phosphorylation
PBMCs: Peripheral blood mononuclear cells
PMA: Phorbol myristate acetate
PPP: Pentose phosphate pathway
ROS: Reactive oxygen species
TCA cycle: Tricarboxylic acid cycle
T_eff_: Effector T cells
T_reg_: Regulatory T cells
T_m_: Memory T cells
